# Linezolid for resistant Gram-positive bacterial infections in children under 12 years: A meta-analysis

**DOI:** 10.1515/med-2022-0440

**Published:** 2022-05-26

**Authors:** Qian Wu, Xiaohua Xu, Mingqing Tian, Jianyang Jiang

**Affiliations:** Department of Respiratory and Critical Care Medicine, People’s Hospital of Quzhou, Quzhou 324000, Zhejiang Province, China

**Keywords:** Gram-positive bacterial infections, pneumonia, methicillin-resistant *Staphylococcus aureus*, linezolid, pediatrics, vancomycin

## Abstract

Both linezolid and vancomycin have good efficacy in the treatment of resistant Gram-positive bacterial infections. This systematic review and meta-analysis aimed to compare the efficacy and safety of linezolid vs vancomycin for the treatment of resistant Gram-positive bacterial infections in children under 12 years.

Five randomly controlled trials involving 638 children that were treated with linezolid and vancomycin for resistant Gram-positive bacterial infections were searched from medical databases. Meta-analysis showed that linezolid and vancomycin had equivalent efficacies in clinical cure rates in the intent-to-treat population (95% confidence interval [CI] 0.88, 2.09) and microbiologically evaluable patients (95% CI: 0.46, 2.47). Linezolid and vancomycin also had equivalent pathogen eradication rates for *Staphylococcus aureus* (95% CI: 0.31, 4.81), methicillin-resistant *S. aureus* (95% CI: 0.36, 5.34), *Enterococcus faecalis* (95% CI: 0.32, 8.76), and coagulase-negative *Staphylococci* (95% CI: 0.43, 4.01). Vancomycin resulted in a higher incidence of alanine aminotransferase increase (95% CI: 0.37, 0.97), red man syndrome (95% CI: 0.01, 0.28), and rash (95% CI: 0.11, 0.73) than linezolid. Clinically, linezolid had a superior safety to vancomycin for resistant Gram-positive infections.

Linezolid might be prescribed for the treatment of resistant Gram-positive bacterial infections in children under 12 years.

## 1 Introduction

Gram-positive pathogens are the most common causes of nosocomial infections in infants and children. They cause a high morbidity of hospital-acquired pneumonia, bacteremia, and mortality [1,2]. The pathogenic bacteria causing pneumonia mainly include coagulase-negative *Staphylococci* (CoNS), *Staphylococcus aureus*, *Streptococcus pneumoniae*, and methicillin-resistant *S. aureus* (MRSA) [1–4]. The emergence and increased frequency of drug-resistant Gram-positive bacteria, including MRSA and vancomycin-resistant *Enterococci* (VRE), are becoming increasing problems for the treatment of nosocomial infections in pediatrics.

Vancomycin is a well-tolerated and effective glycopeptide antibiotic and is the first choice treatment for late-onset sepsis due to resistant *Staphylococci* by neonatologists [1,5,6]. Vancomycin-containing regimens are frequently prescribed for infections caused by multidrug-resistant Gram-positive organisms [3]. However, the emergence of VRE and higher incidence of adverse events are challenging its prescription [5,7,8]. For instance, vancomycin causes idiosyncratic drug actions, including red man syndrome, increased liver enzyme activity, and nephrotoxicity in neonates [4,7,8].

Linezolid is a bacterial protein synthesis inhibitor [9,10]. Linezolid, as the first new thiazolidinone antibacterial drug, has a unique mode of action. It binds to the bacterial 50S ribosomal subunit to prevent the formation of the 70S initiation complex and inhibit protein synthesis in bacteria [9,10]. Linezolid was approved by the Food and Drug Administration of the United States for marketing and pediatric use in 2002 and was approved in China in August 2007. It is mainly used for hospital-acquired pneumonia, bacteremia, and infections caused by multidrug-resistant Gram-positive pathogens, including MRSA, methicillin-resistant CoNS, and VRE [1–4,11,12]. Additionally, linezolid is well tolerated and as effective as vancomycin for the treatment of Gram-positive bacterial infections [2,11]. It is effective for infections of MRSA and VRE [2].

A large number of randomized controlled trials (RCTs) and review analyses have shown the efficacy and safety of linezolid vs vancomycin for the treatment of Gram-positive bacterial infections in adults [2,13–17]. Some reports proposed that linezolid had a significantly lower frequency of drug-related adverse events in patients from birth to 12 years of age than vancomycin [2,4]. However, there was no systematic analysis for comparing the efficacy and safety of linezolid vs vancomycin for the treatment of resistant Gram-positive bacterial infections in neonates, infants, and children <12 years. This study aimed to evaluate the efficacy and safety of linezolid vs vancomycin for the treatment of resistant Gram-positive bacterial infections and to provide medical evidence for pediatricians or neonatologists.

## 2 Materials and methods

### 2.1 Ethics statement

This study was a systematic review to compare the efficacy and safety of linezolid and vancomycin in treating Gram-positive bacterial infections. Neither animal nor human experiments were performed by any one of the authors, and therefore ethics committee approval was not applicable. This study was designed, conducted, and performed following the guidelines of Preferred Reporting Items for Systematic Reviews and Meta-Analyses [18].

### 2.2 Literature source and search strategy

RCTs were searched in comprehensive databases, including PubMed, EMBASE, and Cochrane library using the following words: linezolid, pneumonia, and Gram-positive infections. The search strategy was “Linezolid[MeSH Terms]” AND “newborn[MeSH Terms] OR infant[MeSH Terms] OR children[MeSH Terms] OR child[MeSH Terms] OR pediatrics[MeSH Terms] OR adolescent[MeSH Terms].” Eligible clinical studies that were published up to February 2019 and that compared the efficacy and safety of linezolid and vancomycin in pediatric patients (<12 years) with Gram-positive bacterial infections were included. Additional trials were searched in the reference lists of the review articles and included studies.

### 2.3 Study selection

Eligible clinical trials were selected independently by two authors. Trials were included if they met the following inclusion criteria: (1) RCTs involving pediatric patients (<12 years) with resistant Gram-positive bacterial infections; and (2) patients in the treatment group were treated with linezolid, and patients in the control group were treated with vancomycin. We put no restrictions on race and publication year. Trials were excluded if they were (1) published in non-English; (2) literature duplications, reviews, and case reports; and (3) trials that treated patients in the treatment group with other antibacterial agents in addition to linezolid or treated patients in the control group with other antibacterial agents in addition to vancomycin.

### 2.4 Data extraction

The primary outcomes were the clinical cure rate and pathogen eradication rate. Clinical cure was defined as the disappearance or decrease in main clinical symptoms and pulmonary signs at the end of treatment or the test-of-cure follow-up visit. The safety profiles (adverse events) of linezolid and vancomycin in pediatric patients with Gram-positive infections were extracted.

### 2.5 Assessment of trial quality

Trial quality was assessed using the five-point Jadad scoring tool [19,20], which consists of five items and each item contributes one point to the total score. Trials scoring ≥3 and ≤2 were deemed to be high and low quality, respectively. Two authors assessed quality independently. Discussion or adjudication by a third reviewer was required to resolve disagreements. Publication bias was not assessed because of the small number of included studies.

### 2.6 Statistical analysis

Meta-analysis was performed using Reviewer Manager 5.1 software (RevMan, Copenhagen: The Nordic Cochrane Centre, The Cochrane Collaboration, 2011). The statistical heterogeneity of data across included trials was assessed by the *Q* test and quantified with the *I* ^2^ statistic test. Data of *P* < 0.10 and *I* ^*2*^> 50% were defined as significantly heterogeneous, while data of *P* > 0.10 and *I* ^*2*^< 50% were significantly homogeneous. Meta-analysis was performed with the fixed-effects model due to the significant data homogeneity across the included trials. For meta-analysis of dichotomous outcomes, odds ratios (ORs) and 95% confidence intervals (CIs) were calculated using the Mantel–Haenszel method. Significant differences in efficacy and safety outcomes between linezolid and vancomycin were indicated as *P* < 0.05.

## 3 Results

### 3.1 Study selection

**Figure 1:**
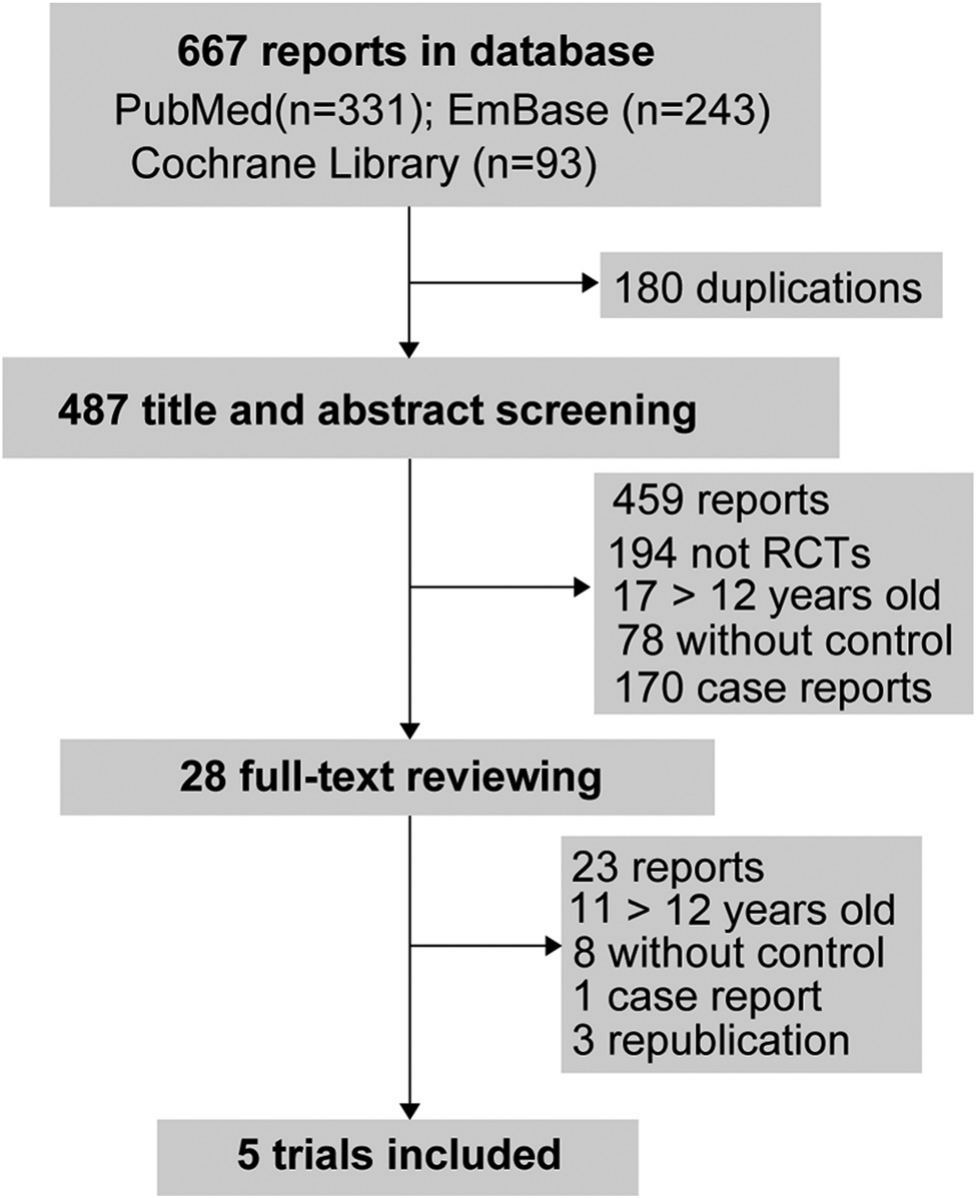
The flow diagram of study selection processing.

The search in medical databases generated 667 reports. After removing duplications (*n* = 180) and screening for title, abstract, and full-text, five trials were included (Figure 1 and Table 1)[1,2,3,4,12].

**Table 1:** Baseline characteristics of the five included studies

Author (year)	Study type	No of patients (ITT)	Age (median)	Type of infection	Organism(s)	Clinical/microbiological efficacy	Jadad score
Kaplan et al., 2003 [2]	RCCT Phase III Open label	219–102	0–11 years (1.8 years)	Nosocomial pneumonia; cSSSIs Bacteremia Systemic infections	MSSA; MRSA; *S. pyogenes*; *S. pyogenes*; CoNS; *Enterococcus* spp.	Clinical success: 89.3% LZD, 84.5% Van microbiological success: MSSA: 95% LZD, 94% Van MRSA: 88% LZD, 90% Van; MR-CoNS: 85% LZD, 83% Van	3
Jantaush et al., 2003 [1]	RCCT; Phase III Open label (subset analysis)	104–48	<12 years (1.15 years); <12 years (1.2 years)	Bacteremia and HAP	*S. aureus*; CoNS; *Enterococcus* spp.	Clinical success: Bacteremia: 84.8% LZD, 80% Van Pneumonia: 90% LZD, 100% Van Microbiological eradication: HAP: 100% LZD, 100%, Van Catheter-related bacteremia: CoNS: 81.8% LZD, 75% Van; Bacteremia: CoNS: 90% LZD, 75% Van	3
Deville et al., 2003 [3]	RCCT; Phase III; Open label (subset analysis)	43–20	0–90 days (18 days); 0–90 days (36 days)	Nosocomial pneumonia; cSSSIs; bacteremia	MSSA; MRSA; CoNS; *Enterococcus* spp.	Clinical success: 84.4% LZD, 76.9% Van Microbiological eradication: CoNS 88% LZD, 100% Van	3
Kaplan et al., 2003 [4]	RCT, multinational, multicenter study	20–14	<12 years	Pneumonia, bacteremia or complicated SSSI	MRSA	Clinical success: 94.1% LZD, 90.0% Van; Microbiological eradication: CoNS 88.2% LZD, 90.0% Van	4
Shibata et al., 2018 [12]	RCT, multicenter	32–36	35 days (range: 4–472)	NICU	Gram-positive infections	Microbiological eradication: 90.6% LZD, 72.2% Van	4

ITT, intention to treat; LZD, linezolid; Van, vancomycin; q8h, every 8 h; MRSA, methicillin-resistant *Staphylococcus aureus*; MSSA, methicillin-susceptible *S. aureus*; MR-CoNS, methicillin-resistant coagulase-negative *Staphylococci*; RCCT, randomized comparator controlled trial; HAP, hospital-acquired pneumonia; cSSSI, complicated skin/skin structure infection.

### 3.2 Trial characteristics

The five intent-to-treat trials involved 638 children with resistant Gram-positive bacterial infections. Four studies were published by the same research team on children (<12 years old) treated with linezolid and vancomycin for 10–28 days [1–4]. All five trials were of high quality (Jadad score: 3–4; Table 1). Four trials reported the clinical cure rate [1–4] and microbiological eradication rate [1–4]. Five trials [1–4,12] reported the safety of linezolid and vancomycin for resistant Gram-positive infections in infants and neonates (4–472 days; Table 2).

**Table 2:** Safety assessment for treatment of resistant Gram-positive infections in children

Adverse events	Study	Linezolid		Vancomycin		*I* ^2^ (%)	*P*	OR (95% CI)	*P*
		Events	Total	Events	Total				
Diarrhea	[1–3,12]	14	379	10	178	0	0.86	0.66 (0.18,69.14)	0.34
Nausea	[1,2]	5	316	0	145	0	0.86	2.76 (0.34,22.70)	0.34
Vomiting	[1,2,12]	18	348	14	181	0	0.62	1.15 (0.62,2.12)	0.67
Rash	[1,2,4]	5	336	10	159	51	0.13	0.29 (0.11,0.73)	0.009
Anemia	[1–4]	7	379	2	178	0	0.98	1.33 (0.36,4.88)	0.67
Red man syndrome	[2,4]	0	233	13	113	0	0.45	0.04 (0.01,0.28)	0.001
Abnormal hematology									
Hemoglobin	[1–3,12]	68	386	27	197	32	0.22	1.27 (0.78,2.08)	0.34
White blood cell count	[1–3,12]	43	386	21	197	0	0.52	0.92 (0.52,1.60)	0.76
Neutrophil count	[1–3,12]	22	375	9	192	0	0.90	1.20 (0.54,2.68)	0.66
Platelet count	[1–3,12]	59	386	34	197	0	0.97	0.86 (0.54,1.38)	0.53
Chemistries									
Alanine aminotransferase increase	[1–3,12]	34	379	27	194	0	0.61	0.60 (0.37,0.97)	0.04
Total bilirubin	[1–3,12]	33	376	11	191	0	0.82	1.50 (0.78,2.87)	0.22
Creatinine	[1–3,12]	10	387	2	197	0	0.50	1.90 (0.48,7.45)	0.36

OR, odds ratio; CI, confidential interval.

### 3.3 Efficacy in the clinical cure rate

**Figure 2:**
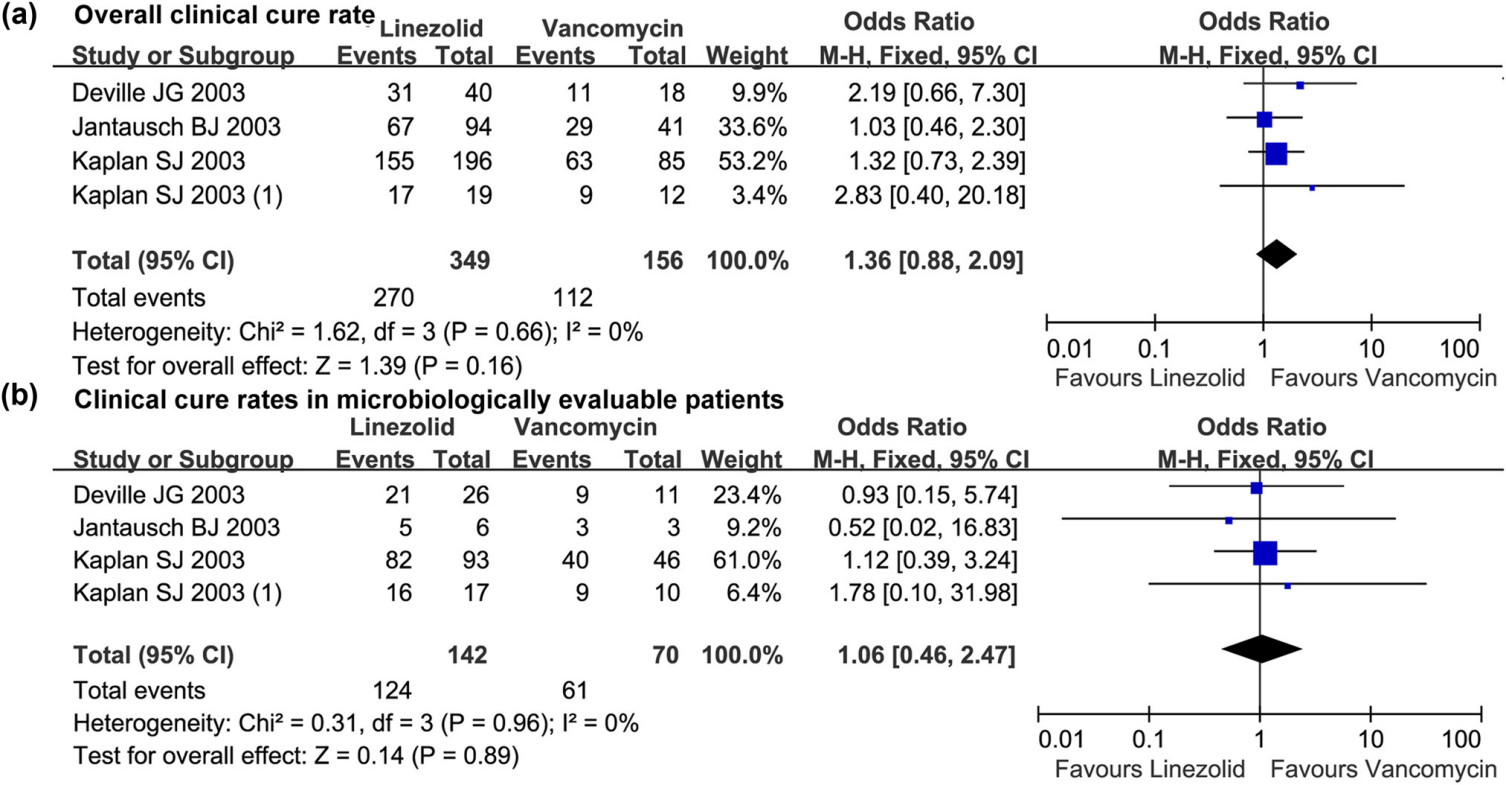
The forest plot of the clinical cure rate of linezolid vs vancomycin in children (<12 years) with resistant Gram-positive bacterial infections. (a) and (b) The comparative overall clinical cure rate and clinical cure rates in microbiologically evaluable patients treated with linezolid vs vancomycin in the treatment of resistant Gram-positive bacterial infections in children under 12 years. M-H, Mantel-Haenszel; CI, confidential interval.

The clinical cure rate data across trials were not heterogeneous (*I* ^*2*^ = 0%, *P* > 0.10). Meta-analysis showed that there was no statistical difference in the overall clinical cure rate between linezolid and vancomycin (OR = 1.36, 95% CI: 0.88, 2.09; Figure 2a) and clinical cure rate in microbiologically evaluable patients (OR = 1.06, 95% CI: 0.46, 2.47; Figure 2b).

### 3.4 Efficacy in the pathogen eradication rate

**Figure 3:**
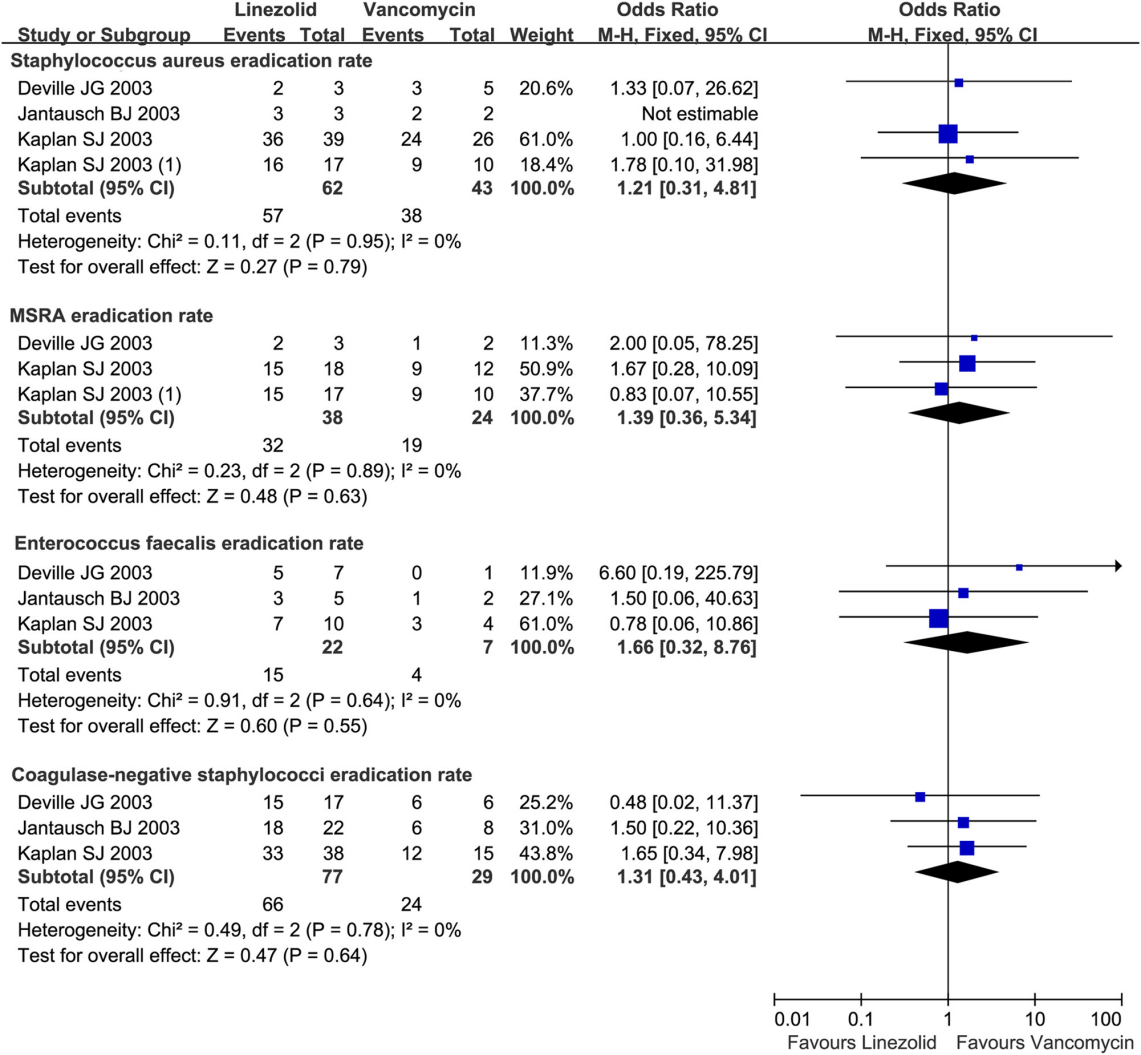
Pathogen eradication rate of linezolid vs. vancomycin in children (<12 years) with resistant Gram-positive bacterial infections. Pathogen eradication rate for *S. aureus*, MRSA, *Enterococcus faecalis*, and CoNS in microbiologically evaluable patients treated with linezolid vs vancomycin for the treatment of resistant Gram-positive bacterial infections in children under 12 years. M-H, Mantel-Haenszel; CI, confidential interval.

The pathogen eradication rate data were not heterogeneous across four trials (*I* ^*2*^ = 0%, *P* > 0.10). A meta-analysis showed that linezolid and vancomycin achieved equivalent efficacies in the eradication rate for *S. aureus* (OR = 1.21, 95% CI: 0.31, 4.81), MRSA (OR = 1.39, 95% CI: 0.36, 5.34), *Enterococcus faecalis* (OR = 1.66, 95% CI: 0.32, 8.76), and CoNS (OR = 1.31 95% CI: 0.43, 4.01; Figure 3) in microbiologically evaluable patients.

### 3.5 Adverse events

**Figure 4:**
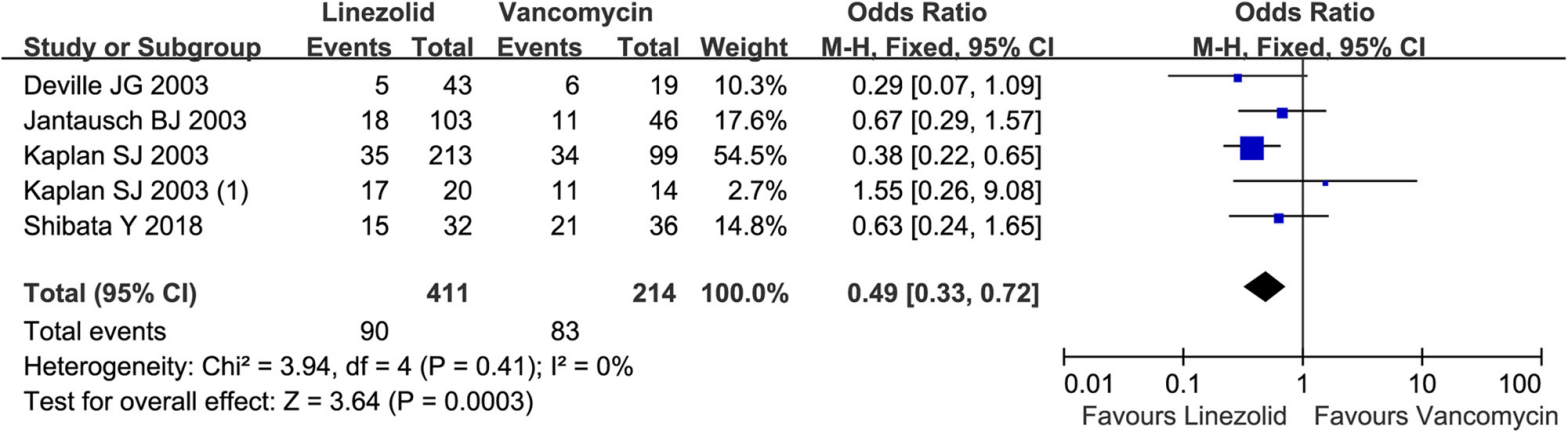
Total adverse event rate by linezolid vs. vancomycin in the treatment of resistant Gram-positive bacterial infections in children <12 years. M-H, Mantel-Haenszel; CI, confidential interval.

Totally, linezolid treatment had a lower frequency of adverse events in children with resistant Gram-positive bacterial infection than vancomycin (90/411 vs 83/214; OR = 0.49, 95% CI: 0.33, 0.72; Figure 4). The subgroup analysis indicated that linezolid and vancomycin achieved equivalent frequencies of diarrhea (95% CI: 0.18, 69.14), nausea (95% CI: 0.34, 22.70), vomiting (95% CI: 0.62, 2.12), anemia (95% CI: 0.36, 4.88), and abnormal laboratory hematology values (including hemoglobin, white blood cell count, neutrophil count, and platelet count), total bilirubin (95% CI: 0.78, 2.87), and creatinine (95% CI: 0.48, 7.45; Table 2). Meta-analysis showed that vancomycin contributed to a higher incidence of alanine aminotransferase increase (95% CI: 0.37, 0.97), red man syndrome (95% CI: 0.01, 0.28), and rash (95% CI: 0.11, 0.73; Table 2) than linezolid.

## 4 Discussion

Our present study confirmed that vancomycin and linezolid had equivalent efficacies against resistant Gram-positive bacterial infections in children under 12 years. In view of safety, vancomycin generated a higher frequency of adverse events, including rash, red man syndrome, and an increase in alanine aminotransferase, than linezolid. These results confirmed that linezolid had a high efficacy and safety in the treatment of resistant Gram-positive bacterial infections in children under 12 years.

Linezolid inhibits protein synthesis and the formation of ribosomal subunit in bacteria [9,10]. It has strong antibacterial activity against drug-resistant *S. aureus* and good permeability in lung tissue [21,22]. Jacqueline et al. [21] showed that linezolid could reduce proinflammatory cytokine tumor necrosis factor α and neutrophil infiltration in a mouse model of MRSA-induced pneumonia. They also showed that linezolid presented a decreased endothelial permeability at 48 h postinfection, while vancomycin resulted in a time-dependent increase of endothelial permeability. This study might indicate that linezolid had superior efficacy against vancomycin in the treatment of MRSA pneumonia [21]. Linezolid also decreased the incidence of nephrotoxicity and adverse events vs vancomycin in the treatment of Gram-positive bacterial infections [4,16,23]. Our present study confirmed that linezolid caused a lower incidence of adverse events than vancomycin particularly in rash, red man syndrome, and abnormal increase in alanine aminotransferase.

Our present study confirmed that linezolid and vancomycin had equivalent efficacies in the treatment of Gram-positive bacterial infections. This finding was in line with the other systematic reviews that were previously reported by Ioannidou et al. [14] and Garazzino and Tovo [24]. A study by Li et al. [23] proposed that the efficacy of linezolid was superior against vancomycin in the treatment of infections caused by MRSA. Liang et al. [16] also revealed that linezolid had a superior clinical and microbiological outcome to vancomycin in skin and soft-tissue infections caused by *S. aureus*. Both the studies found that linezolid presented a better eradication rate than vancomycin in microbiologically evaluable adult patients [16,23]. The result in our study showed that there were no differences in clinical cure rates in microbiologically evaluable and clinically evaluable patients (<12 years) between linezolid and vancomycin. This result was consistent with that reported by Ioannidou et al. [14]. The sample size and patients’ age in these comparisons might be responsible for the differences between these studies.

There is increasing evidence showing the emergence of linezolid-resistant *S. aureus* during the treatment of infections, as well as the co-emergence of linezolid-resistant *S. aureus* and *Enterococcus faecium* in a patient with MRSA pneumonic sepsis [25–28]. Sánchez-García et al. found a clinical outbreak of linezolid-resistant *S. aureus* in ventilator-assisted pneumonia and bacteremia [29]. Toh et al. identified that the acquired linezolid resistance in a hospital MRSA strain was associated with the presence of the *cfr* gene [27]. The *cfr* gene is linked to the *ermB* gene, which confers resistance to all the clinically relevant antibiotics that target the large ribosomal subunit in bacteria [27]. Besier et al. [26] also identified a mutation in the 23S rRNA gene in *S. aureus* that conferred linezolid resistance. The increasing emergence of linezolid-resistant *S. aureus* suggested that new antibiotics are in demand in the treatment of nosocomial infections.

Two limitations were included in this present study. First, the sample size for these comparisons was small (*n* = 638) and studies with larger cohorts should be performed. Second, only five studies were included, and four [1–4] were published by the same research team. Accordingly, this study might reflect the situation of a local hospital. Third, our results showed that there was no difference in the efficacy between linezolid and vancomycin in treating Gram-positive bacterial infections in children under 12 years. However, our present analysis showed that linezolid had a superior safety to vancomycin for resistant Gram-positive bacterial infections. Patients who received linezolid had lower incidence rates of rash, red man syndrome, and alanine aminotransferase increase than vancomycin.

## 5 Conclusion

This systematic review suggested the efficacy and safety of linezolid in the treatment of resistant Gram-positive bacterial infections in children <12 years. Linezolid might be prescribed safely by neonatologists and pediatricians in the treatment of Gram-positive bacterial pathogens. Further studies providing evidence with a larger size of patients should be performed to validate the efficacy of linezolid.

## Abbreviations

CI: confidence interval
CoNS: coagulase-negative *Staphylococci*
FDA: Food and Drug Administration
MRSA: methicillin-resistant *S. aureus*
OR: odds ratio
RCT: randomized controlled trial
VRE: vancomycin-resistant enterococci
